# Extreme Radiation Sensitivity of Ultra-Low Loss Pure-Silica-Core Optical Fibers at Low Dose Levels and Infrared Wavelengths

**DOI:** 10.3390/s20247254

**Published:** 2020-12-17

**Authors:** Adriana Morana, Cosimo Campanella, Jeoffray Vidalot, Vincenzo De Michele, Emmanuel Marin, Imène Reghioua, Aziz Boukenter, Youcef Ouerdane, Philippe Paillet, Sylvain Girard

**Affiliations:** 1 UJM, CNRS, IOGS, Laboratoire Hubert Curien, University Lyon, UMR 5516, 18 rue Prof. B. Lauras, F-42000 Saint-Etienne, France; adriana.morana@univ-st-etienne.fr (A.M.); cosimo.campanella@univ-st-etienne.fr (C.C.); jeoffray.vidalot@univ-st-etienne.fr (J.V.); vincenzo.demichele@univ-st-etienne.fr (V.D.M.); emmanuel.marin@univ-st-etienne.fr (E.M.); imene.reghioua@univ-st-etienne.fr (I.R.); aziz.boukenter@univ-st-etienne.fr (A.B.); ouerdane@univ-st-etienne.fr (Y.O.); 2 CEA, DAM, DIF, F-91297 Arpajon, France; philippe.paillet@cea.fr

**Keywords:** radiation monitor, fiber sensors, optical fibers, radiation effects

## Abstract

We report here the response of a commercial ultra-low loss (ULL) single-mode (SM) pure silica core (PSC) fiber, the Vascade EX1000 fiber from Corning, associated with 0.16 dB/km losses at 1.55 µm to 40 keV X-rays at room temperature. Today, among all fiber types, the PSC or F-doped ones have been demonstrated to be the most tolerant to the radiation induced attenuation (RIA) phenomenon and are usually used to design radiation-hardened data links or fiber-based point or distributed sensors. The here investigated ULL-PSC showed, instead, surprisingly high RIA levels of ~3000 dB/km at 1310 nm and ~2000 dB/km at 1550 nm at a limited dose of 2 kGy(SiO_2_), exceeding the RIA measured in the P-doped SM fibers used for dosimetry for doses of ~500 Gy. Moreover, its RIA increased as a function of the dose with a saturation tendency at larger doses and quickly recovered after irradiation. Our study on the silica structure suggests that the very specific manufacturing process of the ULL-PSC fibers applied to reduce their intrinsic attenuation makes them highly vulnerable to radiations even at low doses. From the application point of view, this fiber cannot be used for data transfer or sensing in harsh environments, except as a very efficient radiation detector or beam monitor.

## 1. Introduction

Optical fiber manufacturers are increasingly interested in developing single-mode waveguides with ultra-low loss at telecom wavelengths in order to guarantee the infrared (IR) signal transmission along hundreds of kilometers in submarine networks, minimizing the need for costly optical repeaters. A few years ago, most commercial Telecom-grade fibers had a Ge-doped silica core and a cladding in pure or slightly doped silica such as the Corning^®^SMF-28TM, which is the standard for data transfer and today has been mainly replaced by Corning^®^SMF-28e+ [1]. However, such optical fibers (OFs) have typical attenuation levels slightly above 0.2 dB/km at 1550 nm, the Rayleigh scattering being the main origin of these optical losses. Nowadays, among all the possible chemical compositions for the optical fibers, the ones based on pure silica core (PSC), sometimes doped with traces of fluorine, are the most promising to further reduce the optical attenuation at this wavelength. Today, the limit is 0.14 dB/km, very close to the theoretical limit for Rayleigh scattering losses [2]. In this case, the fiber cladding must also be doped with fluorine to decrease the refractive index and to ensure the light guidance by total internal reflection. The principal cause for transmission decrease today in PSC OFs is the Rayleigh light scattering resulting from SiO_2_ density fluctuations in the fiber core and cladding and at their interface. Therefore, efforts are mandatory to achieve a very specific manufacturing process, allowing the decrease in glass density fluctuation and then the associated losses in order to achieve the so-called ultra-low loss (ULL) optical fibers [2].

Interestingly, the F-doped and PSC fibers have also been identified as the most promising waveguides for data transfer or multi-parameter (temperature, strain, pressure, liquid level…) point or distributed sensing [3] in severe environments. Usually, these applications also work at telecom wavelengths to benefit from the technologies developed for telecommunications. Some of these sensors have to operate in harsh environments that are associated with steady-state radiation constraints such as the ones encountered in nuclear power plants, space, or at high energy physics facilities [4]. In this case, it becomes of primary importance to understand how these environmental constraints could affect their performances and, if necessary, identify mitigation techniques to the observed degradation mechanisms. Indeed, in the presence of radiation, three main phenomena have been identified that are susceptible to affecting the reliability of fiber-based devices or systems. The most studied is called radiation-induced attenuation or RIA. RIA consists of the increase of the fiber attenuation at the operating wavelength(s) of the application. RIA is caused by the appearance of absorption bands in the transmission window (from the ultraviolet to the infrared domains) of the optical fibers. These absorption bands are caused by radiation induced point defects created in the silica glass matrix by either ionization or knock-on processes. RIA directly affects the fiber transmission properties [4], altering the quality of data links or degrading the achievable sensing length of single-ended or double-ended distributed sensors [5]. The amplitudes and kinetics of the RIA have been shown to depend on many parameters intrinsic to the fiber itself (composition, manufacturing process …) and to the irradiation conditions (dose, dose rate, temperature of irradiation). It is then very difficult to avoid the RIA, but it is possible to limit its impact by developing radiation hardening strategies either at the device level (optimizing its composition to limit the RIA) or at the application level (for example, by changing the operating wavelength) [5]. The other two phenomena, the radiation-induced emission and the radiation-induced compaction [5], can usually be more easily managed for environments associated with low dose-rates and doses below 100 kGy(SiO_2_) [4] such as the one considered in this article.

We focused our attention on the RIA in an ultra-low loss pure silica core (ULL-PSC) optical fiber. From the existing literature, one could assume a good fiber radiation resistance in the IR part of the spectrum compared to other classes of optical fibers. The Ge-doped ones are usually notably quite sensitive to radiation [4] and the phosphosilicate ones are known to be so highly radiation sensitive that they are today used for distributed dose monitoring (i.e., at CERN, the European Organization for Nuclear Research, or in neutron-rich environments) [6,7]. The RIA, indeed, depends on the fiber composition, but also on other parameters such as the fiber treatment (i.e., a pre-loading with H_2_, the irradiation conditions such as particle type, dose, dose-rate, irradiation temperature), and the injected light power, which can bleach radiation-induced point defects [5]. Basically, for pure silica core fibers, the radiation response also depends on the glass fictive temperature and the presence of impurities such as hydroxyl groups and chlorine traces [8,9,10]. As a consequence, it is interesting to investigate how the ULL-PSC fiber optimization in terms of intrinsic attenuation and Rayleigh scattering loss reduction at 1.55 µm could have affected its steady state radiation response in terms of RIA.

The dose and dose-rate dependence of the RIA of this ULL-PSC fiber was investigated and compared with other classes of fibers under X-rays. It is, indeed, known that these latter produce the same effects induced on optical fibers by γ-rays at the same dose and dose-rate, or by neutrons at low fluences, where the main effect is the ionization [5].

To support the RIA data analysis, an additional technique (Raman spectroscopy) was applied in order to compare the disorder degree of the ULL-PSC fiber glass matrix to the ones of other PSC fibers with the objective to find a relationship between the glass structural properties and the observed high radiation sensitivity.

## 2. Materials and Methods

### 2.1. Investigated Samples

**Figure 1:**
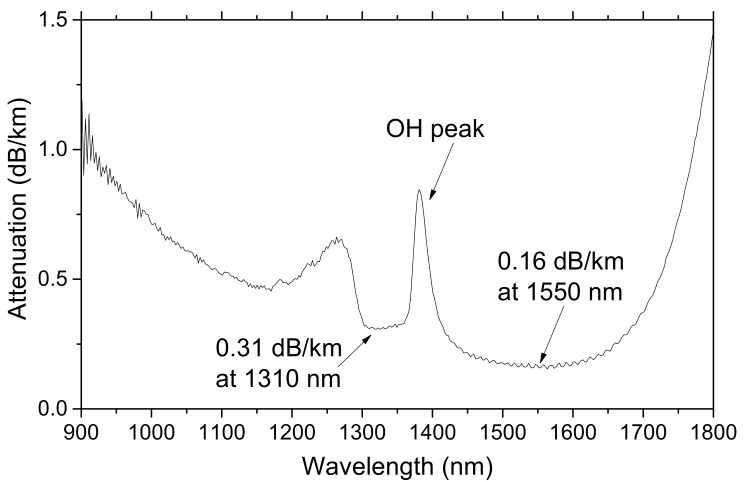
Spectral attenuation of the tested ultra-low loss pure silica core (ULL-PSC) fiber before irradiation.

The radiation response of a commercially-available ULL-PSC single mode optical fiber, the Vascade EX1000 fiber from Corning, was investigated here. This fiber is characterized by ultra-low losses at 1.55 µm, below 0.16 dB/km, as reported in its attenuation spectrum given in Figure 1 and obtained through cutback measurements on the unirradiated tested sample. This technique consists of acquiring the transmission spectra of a long and a short length of the sample, $I_{L}\left(\lambda \right)$ and $I_{l}\left(\lambda \right)$, respectively, without changing the injection conditions. The spectral attenuation is then obtained as:

(1) $$attenuation\;\left(\lambda \right)=-\frac{10}{\left(L-l\right)}\log_{10}\left(\frac{I_{L}\left(\lambda \right)-I_{noise}\left(\lambda \right)}{I_{l}\left(\lambda \right)-I_{noise}\left(\lambda \right)}\right),$$

where $I_{noise}\left(\lambda \right)$ is the background acquired with the source off.

**Figure 2:**
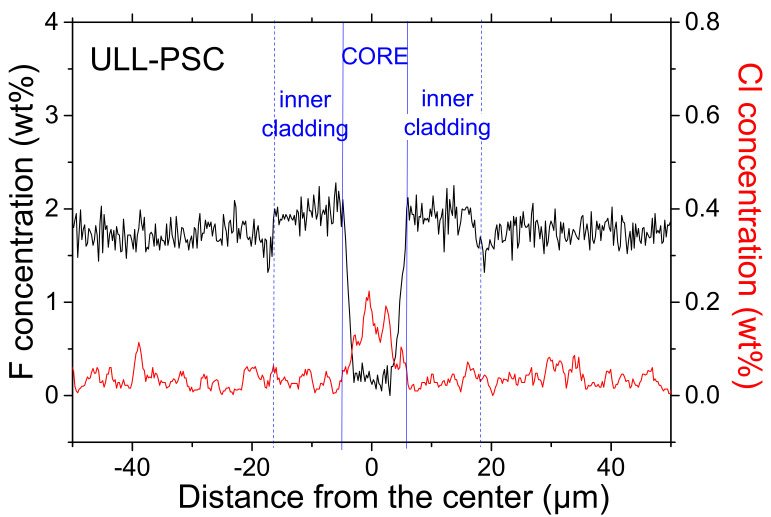
Radial distributions of chemical elements measured by EDX along the ULL-PSC fiber diameter: Fluorine (left vertical axis) and chlorine (right vertical axis). The continuous blue lines limit the fiber core zone and the dashed ones mark the presence of an inner cladding (where the evanescent part of the fundamental mode propagates) revealed by those measurements.

The attenuation spectrum of Figure 1 highlights the presence of an attenuation peak at around 1380 nm (band amplitude of 0.85 dB/km) associated with Si–OH groups. Using the data of [11], the concentration of hydroxyl groups was estimated to be about 14 ppb. The observed asymmetric band around 1280 nm was due to the transition between the unimodal and bimodal propagation regimes and could be seen as an artifact related to the cut-back measurement. The fiber is single-mode at both 1310 nm and 1550 nm. This silica-based fiber contains fluorine as the main dopant used to create the refractive index profile. It also contains some traces of chlorine impurity, related to the fiber manufacturing process, as shown in Figure 2, which revealed the composition of the tested sample.

These chemical analysis results were obtained on the fiber with a field emission scanning electron microscope (JSM 7100f JEOL) equipped with energy dispersive X-ray (EDX) detector (X-Max 80, Oxford, UK) at the Nova Gorica University (Nova Gorica, Slovenia). The analysis confirmed the presence of fluorine traces in the fiber core, whose diameter was 10 µm, with a F-concentration of around 2% in the fiber inner cladding where the evanescent part of the fundamental LP01 mode is propagating.

The ULL-PSC OF radiation-response was compared with the ones of other single-mode fiber classes: the standard germanosilicate SMF28e+ from Corning, a radiation-sensitive P-doped fiber discussed in [6], and two other commercial F-doped samples intended for operation in harsh environments, hereafter indicated as F1 and F2, from two different manufacturers: iXblue Photonics (Lannion, France) and Fujikura (Tokyo, Japan). These two radiation-resistant optical fibers are manufactured using two different preform fabrication methods: surface plasma chemical vapor deposition (SPCVD) and outside vapor deposition (OVD). These two F-doped fibers have pristine attenuation (before irradiation) at 1550 nm, respectively, of 0.83 dB/km and less than 0.5 dB/km. Moreover, both of them have already been demonstrated to be resistant to steady state irradiations [12,13] and able to be used for Brillouin, Rayleigh, or Raman based sensing up to MGy dose levels [14,15,16,17]. 

### 2.2. Irradiation Tests

**Figure 3:**
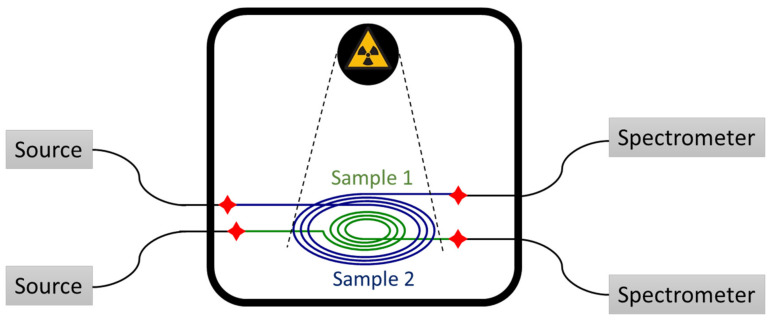
Experimental setup for radiation induced attenuation (RIA) measurements at the MOPERIX X-ray facility. The black continuous lines represent the rad-hardened fibers used to transport the signal.

Different lengths of samples were coiled in a spiral shape, as reported in Figure 3, and irradiated at 175 mGy(SiO_2_)/s dose-rate up to 2 kGy(SiO_2_) total ionizing dose (TID) with the MoperiX facility. This is a tungsten X-ray tube operated at 100 kV, delivering photons with mean energy of ~40 keV at the Laboratoire Hubert Curien (Saint Etienne, France). All the presented results were obtained at room temperature (about 22 °C). The transmission spectra of the different samples were recorded by using two halogen sources (DH2000 from Ocean Optics, Orlando, FL, USA) and two NIR (near infrared) spectrometers (NIR512 or NIRquest from Ocean Optics, working in the spectral range from 850 nm up to 1700 nm or 2100 nm, respectively). For the study of the RIA dependence of the ULL-PSC OF on the dose-rate, the transmission spectra of ~1 m of fiber were recorded with the same halogen source and the same NIR spectrometer (NIRquest from Ocean Optics) at three different dose-rates (10, 40, and 175 mGy/s) up to 2 kGy TID. For each dose-rate, a new pristine sample was irradiated. The dosimetry was performed with a soft X-ray ionization chamber before each of the irradiation runs with a precision of about 10%.

### 2.3. Raman Spectroscopy

In order to investigate the structural disorder degree of the silica-based optical fibers, the integrated confocal microRaman system Aramis (Horiba Jobin Yvon) at the Laboratoire Hubert Curien (Saint Etienne, France) was used. This was equipped with a He-Cd laser emitting at 325 nm, 3D-microtranslation stages, and a CCD camera, allowing us to acquire Raman spectra on the fiber transverse surface. In particular, the spectra were recorded at the centers of the cores of all three F-doped fibers with a ×40 objective and a confocal hole diameter of 75 μm, giving rise to a spatial resolution of ~5 μm.

## 3. Results

### 3.1. Radiation Induced Attenuation

**Figure 4:**
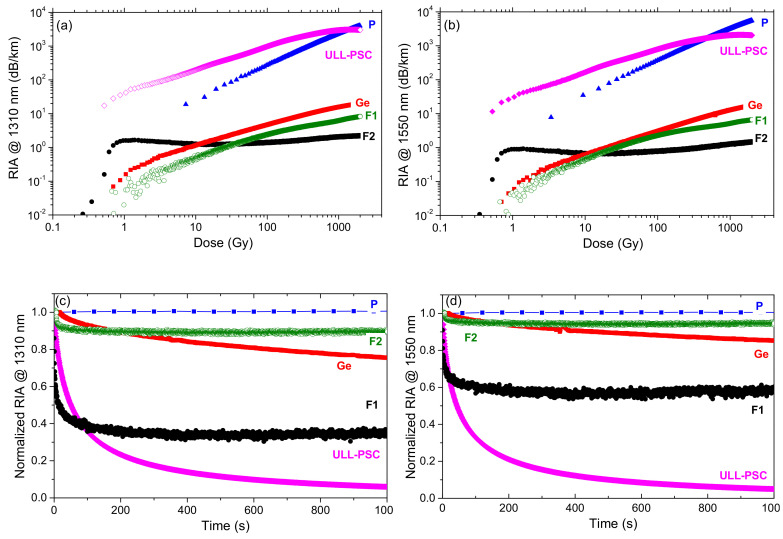
RIA growth kinetics at (**a**) 1310 nm and (**b**) 1550 nm as a function of the dose during the steady state X-ray irradiation (175 mGy/s, room temperature) of the four tested samples: P-doped (blue full triangles), Ge-doped (red full squares), F-doped (green empty circles—F1, black full circles—F2), and ULL-PSC (pink empty diamonds) optical fibers. In (**c**) and (**d**) are illustrated the RIA recovery kinetics after the end of the irradiation for the different fibers at 1310 nm and 1550 nm, respectively.

Figure 4 reports, for the various optical fibers, the growths of the RIA caused by the X-rays at 1310 nm and 1550 nm. The induced losses were recorded up to 2 kGy(SiO_2_) TID at a dose-rate of 175 mGy(SiO_2_)/s.

As already observed in [6], the P-doped OF losses at 1550 nm increased linearly as a function of the dose up to ~500 Gy: up to this TID, the slope was about 4 dB km^−1^ Gy^−1^, whereas the sensitivity coefficient started decreasing at higher doses. The fiber radiation behavior at 1310 nm was very close, with slightly lower RIA level. Moreover, after irradiation, there was no RIA recovery at both wavelengths, as shown in Figure 4c,d. The high radiation sensitivity of the P-doped fibers in the IR domain is well-known and is explained by the unique properties of one of the P-related point defects: the P1 defect. The P1 paramagnetic structure corresponds to a P atom bonded to three oxygen atoms and hosting an unpaired electron [18]. This point defect is associated with an absorption band around 0.79 eV (~1570 nm, FWHM of 0.29 eV), which is mostly responsible for the IR-RIA at both telecommunications wavelengths, even at larger doses than the ones considered in this article [19]. The P1 defect is very stable at room temperature, explaining how no recovery was observed in our test conditions. All of these properties explain that these fibers allow the development of very efficient dosimeters, for example, for monitoring the dose distribution along the CERN accelerators by combining such fibers with reflectometry techniques [20].

As expected from the literature [21], the Ge- and F-doped fibers were less radiation-sensitive than the P-doped one under steady state X-ray irradiation. In this case, RIA at 1550 nm and 1310 nm was below 10 dB/km compared to the losses exceeding 1000 dB/km for the P-doped sample, at the maximum TID. A limited RIA recovery for the Ge-doped sample was observed at the end of the irradiation, below 20%, 1000 s after its stop at the two considered wavelengths. The losses of the Ge-doped sample were then at least two orders of magnitude lower than the P-doped fiber. In the near-IR, the origins of the RIA in the germanosilicate optical fibers were still under investigation. An optical absorption band around 900 nm (1.38 eV) has recently been observed and associated with a Ge-related defect, named GeY [22]. The RIA contribution at larger wavelengths (non-Gaussian shape), instead, has been tentatively explained by the generation of Ge-related self-trapped holes (Ge-STHs) by analogy with the work of Chernov in pure silica [23]. Ge-STHs and GeY defects are still not sufficient to fully reproduce the measured RIA spectra, at least another source of optical losses around 0.9 eV has to be identified for continuous irradiation [21]. This is also true for pulsed irradiation, even if in this case, a recent study pointed out the STH role on the steady state and transient response of germanosilicate fibers [24,25].

The two F-doped OFs are even more radiation-resistant than germanosilicate optical fibers, as expected [4], independently of the manufacturer, F-doping profile, or the impurities. However, it is worth noticing that these parameters strongly influence the RIA behavior of such radiation hardened optical fibers. Indeed, the F2 sample showed lower RIA than F1 only for doses higher than 10 Gy. The differences in the RIA growth and decay kinetics are probably explained by the fact that these fibers were manufactured with different preform deposition processes. Consequently, they probably contain different types and concentrations of precursor sites for the radiation induced point defects. For these two fibers, the RIA recovery efficiencies also differed, reaching up to 40% about 1000 s after the irradiation end for the F1 fiber.

The ULL-PSC fiber, nevertheless, with its quite comparable composition, exhibits a completely different behavior compared to the other F-doped samples. At low doses, up to ~500 Gy, the RIA was the highest reported among the different tested fiber types including the P-doped sample: induced losses of ~200 dB/km and ~170 dB/km were measured at 1310 nm and 1550 nm at a dose of 10 Gy. At 1 kGy TID, instead, the RIA levels reached values around 3000 and 2000 dB/km, respectively. Such high loss values have never been observed for pure-silica core optical fibers exposed to steady state irradiation in the IR domains and are totally unexpected from the current knowledge about radiation effects on silica-based optical fibers [5]. It also demonstrated that pure-silica core optical fibers can no more be considered as radiation resistant compared to the other classes of doped silica core fibers, even for dose levels as low as those encountered in space applications. In the next sections, spectral measurements of the RIA are performed in order to better characterize the origins of these high RIA levels.

### 3.2. Dose-Rate Dependence

**Figure 5:**
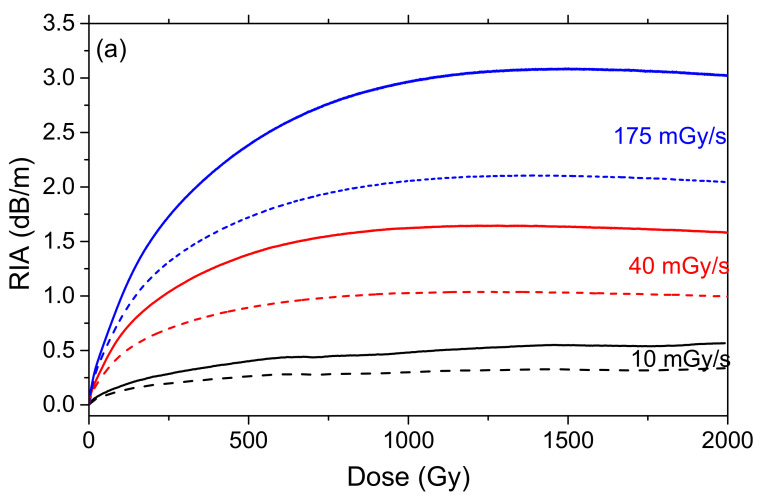

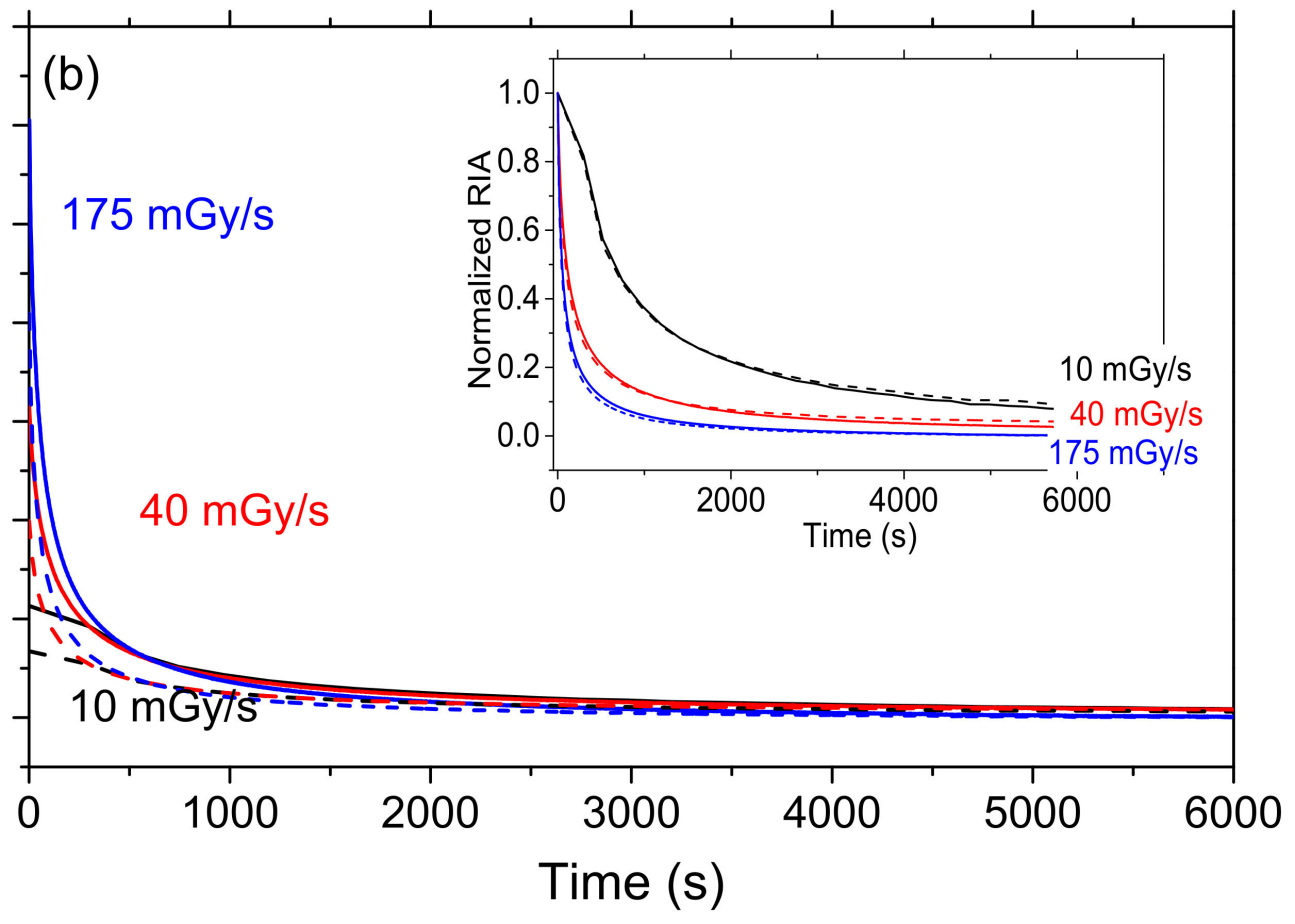
ULL-PSC RIA kinetics (**a**) as a function of the dose during the irradiation and (**b**) as a function of the time during the recovery, at 1310 nm (continuous lines) and 1550 nm (dashed lines), up to TID of 2 kGy, for three dose-rates: 10 (black curves), 40 (red curves), and 175 mGy/s (blue curves—results already reported in Figure 4). In the inset, normalized RIA during the recovery phase.

Dose and dose-rate are known to influence the RIA levels and kinetics in silica-based optical fibers [4]. During the irradiation, the observed RIA growth kinetics result from the competition between the generation of radiation induced defects and their thermal or photo-bleaching [26]. When the dose rate increases, the irradiation duration decreases for a given total ionizing dose. Consequently, induced defects then have less time to recover, and as a consequence, larger RIA, are expected. At the end of the irradiation, the part of the metastable defects not bleached during the irradiation can be thermally or photobleached, resulting in a decrease in the RIA up to a permanent RIA level caused by the point defects stable under the test conditions (temperature, injected light power). To study the radiation induced losses on the ULL-PSC OF and the influence of the dose-rate, spectral RIA measurements as a function of dose and dose-rate were carried out both during and after irradiation, at three different dose-rates (10, 40, and 175 mGy/s) up to 2 kGy TID. All the experiments were done on new fiber samples. Figure 5a reports the RIA kinetics at 1310 nm and 1550 nm as a function of dose (or time) during the irradiation, for the three dose-rates, whereas Figure 5b displays the RIA behavior as a function of time during the recovery after 2 kGy TID.

The RIA at both wavelengths quickly increased during the irradiation and also quickly decreased after it. We measured at 1550 nm (1310 nm) and for a TID of 2 kGy, radiation-induced losses of about 3000 dB/km (2000 dB/km), 1500 dB/km (1000 dB/km), and 500 dB/km (300 dB/km) at dose rates of 175 mGy/s, 40 mGy/s, and 10 mGy/s, respectively. It should be noted that the higher the dose-rate, the larger the induced attenuation at the same dose. We could then expect that at higher dose rate, the measured RIA in the ULL-PSC OF could be larger than 3000 dB/km, largely exceeding the IR-RIA of phosphosilicate optical fibers that are dose-rate independent in this range of dose. The recovery kinetics after irradiation also depend on the dose rate: the larger the dose rate, the faster the recovery after irradiation. It is worth noting that, independent of the dose-rate, the induced losses for this ULL-PSC fiber were larger at 1310 nm than at 1550 nm. However, whereas the RIA kinetics differed at both wavelengths during irradiation, during recovery, they had the same trend. Interestingly, regardless of the dose rate, the permanent RIA levels (remaining losses 1000 s after the irradiation ends) were identical for the three samples, at both wavelengths. This suggests that the permanent induced losses are caused by room temperature stable defects differing from the metastable ones responsible for the large transient losses observed during irradiation. Furthermore, the RIA related to these stable defects should be less dose-rate dependent, but dedicated measurements are needed to better characterize this minor contribution to the RIA observed during irradiation.

### 3.3. Origin of the Induced Attenuation

**Figure 6:**
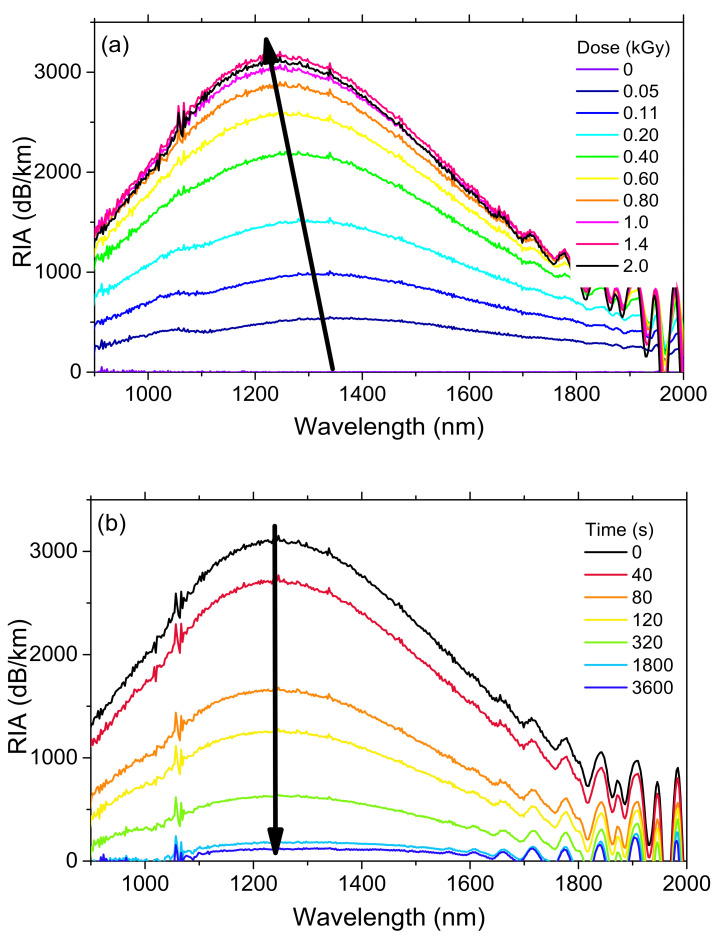
ULL-PSC spectral RIA (**a**) as a function of the dose during the irradiation and (**b**) as a function of the time delay from the irradiation end during the recovery phase, the dose-rate being 175 mGy/s. The arrows indicate the time that elapses.

In order to study the origin of these non-permanent losses, Figure 6a,b show the spectral shape of the radiation-induced attenuation, respectively, at different doses and different time delays from the irradiation end, for the highest investigated dose-rate, 175 mGy/s, at room temperature.

Figure 6 highlights the presence of a large absorption peaked around 1200 nm. This absorption very quickly grows at lower doses and tends to saturate at the largest considered doses (above 1 kGy). This behavior suggests that this absorption could be related to the generation of absorbing defects from precursor sites, already present before irradiation and converted into optically-active defects by the X-rays. If this is the case, after all precursor sites are converted, we could imagine that irradiation at larger doses should reveal a different kinetic for the IR-RIA, related to other classes of point defects. Clearly, this large absorption, never observed at such intensity in the literature, explains the very radiation sensitivity of the ULL-PSC optical fiber. Furthermore, our measurements showed that band peak shifted toward the shorter wavelengths with increasing TID (as illustrated by the black arrow in Figure 6). Such shift was not observed during the fast-post-irradiation RIA recovery. This suggests the presence of more than one absorption band, and possibly that more than one defect is involved in the degradation of the fiber IR transmission.

**Figure 7:**
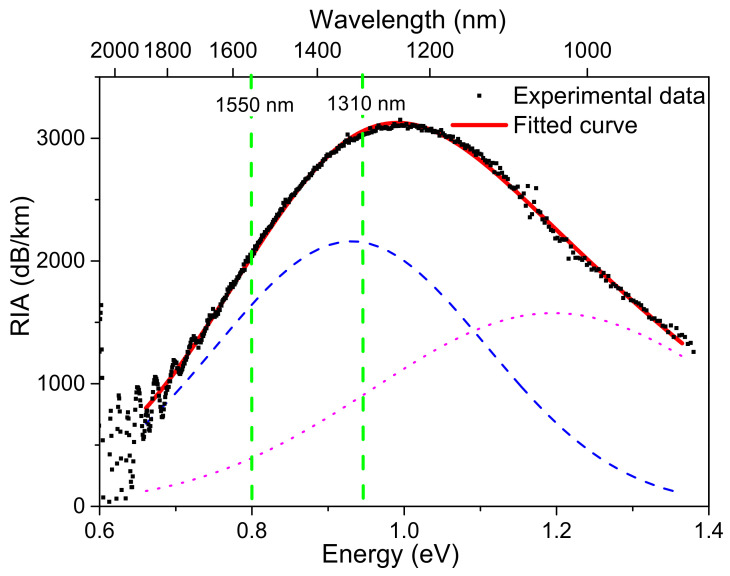
Spectral decomposition of the RIA acquired at the highest dose (2 kGy) and dose-rate (175 mGy/s): experimental data (full black squares), fitted curve (red continuous line), which is the sum of two Gaussian bands (dashed blue and dotted pink lines).

In order to verify this hypothesis, Figure 7 displays an example of spectral decomposition of the measured infrared absorption (as a function of energy *E*), in particular, the one obtained at the maximal TID and the highest dose-rate, as the sum of two Gaussians, described as:

(2) $$RIA\left(E\right)=\overset{2}{\underset{i=1}{\sum }}A_{i}\cdot \exp\left[-\frac{{\left(E-E_{i}\right)}^{2}}{2\;\sigma_{i}^{2}}\right]$$

where *Ai*, *Ei*, and *σi* are the amplitude, the center, and the variance of the *i*-th absorption band, respectively. Moreover, it is worth noticing that *σ* is linked to its full width at half maximum (FWHM) through: $FWHM=2\sqrt{2\ln2}\;\sigma$. The first band is peaked at ~0.93 eV, which corresponds to ~1330 nm (full width at half maximum, FWHM, of ~0.42 eV), and the other centered at ~1.2 eV, that is ~1030 nm (FWHM of ~0.56 eV). It is interesting to note that at very low doses (50 Gy and 110 Gy in Figure 6a), it seems possible to discriminate these two bands directly on the RIA spectra as the one centered at ~1030 nm seems to contribute more to the RIA at the very beginning of the irradiation. More tests will have to be done with varying lengths of samples to better highlight the specific growth and decay kinetics of these two bands with the dose.

An absorption around 1200 nm has already been reported by Kashaykin et al. in γ-irradiated pure silica core fiber samples at TID of 1 kGy (dose-rate being ~1 Gy/s) [27]. By studying the RIA of PSC polarization maintaining fibers, the same authors managed to isolate two contributions: one centered around 0.95 eV (FWHM~0.34 eV) and another peaking at ~1.12 eV (FWHM~0.6 eV) [28]. As the growth and decay kinetics of these bands appear to be close to the ones of self-trapped holes (STHs) (see pioneer papers on this topic [29,30]), these optical signatures were tentatively associated with an inherent STH [28]. Usually in pure-silica-core optical fibers, the closest STHs’ related absorption bands to the IR are the ones associated with the strain-assisted STH_1_ and STH_2_. STH_1_ is a hole trapped on a normal bridging oxygen in the glass network, while STH_2_ is a defect where the hole is rapidly tunneling between two adjacent oxygen atoms. STH_1_ and STH_2_ are associated with absorption bands peaked at 1.63 eV and 1.88 eV, respectively, while the inherent STH_2_ is peaked at lower wavelength, around 2.16 eV [31].

The two bands identified in the ULL-PSC sample were close to the ones observed by Kashaykin et al. [19]. A major difference concerns the RIA level reached at 1.55 µm in the ULL-PSC OF. Indeed, if the losses induced at 1550 nm in the PSC fibers studied by Kashaykin et al. do not exceed 20 dB/km at the TID of 1 kGy [27], the losses here reported are two orders of magnitude higher. This suggests that if STHs are responsible for the observed IR losses in the ULL-PSC sample, the manufacturing process of this fiber should enhance the concentration of precursor sites that will be converted into STHs under X-rays. As it was previously shown that the glass structure properties influence the STH generation under irradiation, in the next section, Raman measurements on the ULL-PSC are performed to compare its glass structure to the ones of the two other F-doped samples.

### 3.4. Self-Trapped Holes (STH) Yield and Structural Disorder

As demonstrated by Tamura et al. [2], the pristine fiber attenuation at 1550 nm, mainly due to Rayleigh scattering losses, can be decreased by reducing the silica glass structure disorder. A parameter describing this latter is the fictive temperature (T_f_), which is defined as the temperature at which the liquid structure is frozen through the glass transition [32]. The fictive temperature can be calculated from the Raman spectra, in particular, increasing the fictive temperature caused a blue shift of the main R band (peaked around 400 cm^−1^ and assigned to symmetric stretching of bridging oxygens in 6-membered rings) and an intensity increase in the D2 band (peaked at 606 cm^−1^ and proportional to the concentration of three-membered ring) [32].

**Figure 8:**
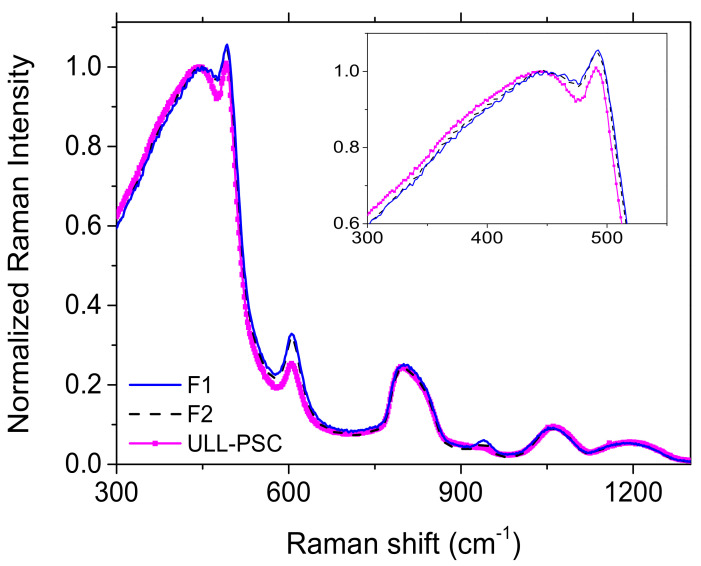
Normalized Raman spectra acquired in the fiber core of the ULL-PSC (continuous pink line with squares), F1 (blue continuous line) and F2 (black dashed line) samples. In the inset, a zoom of the main band.

The Raman spectra acquired in the core center of the three F-doped fibers (F1, F2, and ULL-PSC) and normalized to the main band are reported in Figure 8 and highlight that the structure of the core of the F1 and F2 samples were similar, but differed from the one of the ULL-PSC OF. In particular, the main band frequency and the D2 band intensity were lower in the latter than in the other two fibers, entailing that the ULL-PSC OF had a lower fictive temperature, in agreement with the results of Tamura et al. [2]. Unfortunately, the T_f_ values cannot be quantitatively calculated, since the calibration curve is known only for the very pure silica sample [33] and all three investigated optical fibers had a low F concentration.

The existence of a relationship between the STH generation and the glass structure has also been demonstrated. Yamaguchi et al. were the first to show that STH yield increased with T_f_ increase, at least for the pure silica samples [34]. Furthermore, Wang et al. observed that for samples having the same T_f_, the higher the fluorine concentration, the lower the STH yield [35]. Indeed, both decreasing T_f_ and increasing F concentration led to an increase of the Si–O–Si angle and a reduction of the Si–O bond length [35]. The STH production, instead, is associated with the presence of strained bonds [36], characterized by smaller bond angles and longer bond lengths [35].

All the works reported so far in the literature [8,34,35,36] would have led to the conclusion that the ULL-PSC OFs, as the one here investigated, having lower fictive temperature than the more classical F-doped samples, should be the most radiation-hardened fibers among all the types and that the concentration of the radiation-induced STHs in such fibers should be very low. However, the experimental results, here reported, did not lead to the same conclusion. For the first time, it cannot be stated that decreasing the fictive temperature improved the radiation resistance. Even if the chemical analysis of the ULL-PSC fiber highlights only the presence of Cl and F, other dopants or impurities could be present below the EDX technique detection limit. Indeed, beside Cl and F, even alkali metals such as K, Na, Li, Cs, and Rb are often used to decrease the fictive temperature in very low quantity (~20 ppm wt %) [37,38,39], making them undetectable by the EDX technique. Consequently, we can today only hypothesize that the ULL-PSC OF has probably been co-doped with small amounts of alkali metals to decrease its fictive temperature and its intrinsic optical losses at 1550 nm. Our study revealed that the manufacturing process modifications alter the nature and/or the concentration of precursor sites that are converted into new defects (maybe new types of inherent STHs, to be confirmed), causing very high RIA levels in the IR domain even at low radiation doses. It is worth noticing that the precursors are not directly linked to the alkali metals, but are probably strained bonds. This can be stated since the two bands identified in the ULL-PSC OF have already been observed by Kashaykin et al. [28] after the integration of borosilicate stress-applying rods in their pure silica samples.

## 4. Conclusions

In conclusion, we studied the radiation response of an ultra-low loss PSC fiber, whose intrinsic losses at 1550 nm were ~0.16 dB/km in radiation-free environments. This sample is characterized by a F-doped silica cladding and a pure-silica core, presenting Cl and OH groups as impurities. Despite its composition, such a fiber does not show the classical response of commercial pure-silica core or F-doped fibers that are usually resistant to steady state irradiation and widely used for data transfer or sensing applications. Induced IR losses as high as 3000 dB/km and 2000 dB/km have been measured at 1310 nm and 1550 nm at the limited dose of 2 kGy (dose-rate being 175 mGy/s). Such RIA levels make it impossible to use this fiber for any of the targeted applications needing radiation tolerant waveguides, either for data transfer or distributed sensing. It was also observed that most of the RIA was recovered after the irradiation, highlighting the metastable nature of the radiation induced point defects at the origin of the fiber degradation.

Our online spectral measurements of the RIA during the irradiation run allow us to demonstrate that the excess of optical losses seems related to the generation of two absorption bands in the infrared domain. The first one was centered at 1030 nm (FWHM of 0.56 eV) and the second one at 1330 nm (FWHM of 0.42 eV). Such absorptions make the fiber more radiation sensitive than the P-doped OF at 1550 nm, for doses up to 500 Gy. However, contrary to the latter, which shows no recovery at all after irradiation, the defects at the origin of these absorptions recombine quickly when the irradiation stops. We observed that the losses at 1310 nm or 1550 nm were also strongly dose-rate dependent, at least between 10 and 175 mGy/s: the higher the dose-rate, the larger the saturation value of the losses, but the faster the recovery in percentage terms.

Such a fiber with this completely unexpected radiation-response highlights two important points from the application point of view. First, not all pure silica core fibers are radiation-resistant: their radiation-sensitivity strongly depends on the silica structural properties and dopants. We hypothesize the presence of alkali metals, dopants often used to reduce the silica glass fictive temperature and the pristine losses at 1.55 µm, to be at the origin of the precursor sites (i.e., strained bonds) leading to such absorptions. Second, such fiber radiation sensitivity, and above all the defects at their origin, could give rise to an optical fiber detection system of radiation presence or be useful for beam monitoring. Indeed, this sensor will have two states: one ‘ON’ characterized by high loss level, and one ‘OFF’ with low losses, since the defects appear under the presence of radiation and, once the radiation stops, they quickly disappear, coming back to an attenuation value near the pristine one. However, because of the dose rate dependence, the fiber does not currently appear as very promising for a total ionizing dose sensor, but contrary to the P-doped fiber, the ULL-PSC once seems to work at TID as high as 2 kGy, and not only to 500 Gy. Moreover, more studies are in progress to extend the characteristics of such a sensor and to also understand its limits.

From the material science point of view, the observed bands were tentatively associated with news types of self-trapped holes (STHs) [28]. However, further studies should be performed in order to confirm their origin. Knowing that post mortem analysis techniques such as electron paramagnetic resonance (EPR), photoluminescence, or time-resolved luminescence (PL, TRL) will be difficult to apply regarding the transient nature of these defects at room temperature, one should focus on techniques that can be applied in situ (during irradiation) such as radio-luminescence or online EPR measurements. As prospective studies, it will then be very interesting to investigate the radiation response of this fiber in the UV-near-IR spectral range to determine if this high defect concentration corresponds to an increased absorption in the UV–Visible (i.e., at 475 nm, 574 nm, 660 nm, and 760 nm) wavelengths at which the bands associated with the STH are centered [21], or induces the appearance of additional new absorption bands. Furthermore, it will be very interesting to investigate the effect of varying the temperature of irradiation on the growth and decay kinetics of this IR-RIA.
